# Supplementary material for: Individual, community, and societal drivers of adolescent peer violence in Asella, Ethiopia: A school-based cross-sectional study utilizing the socio-ecological model
Source: PLoS One. 2026 May 28;21(5):e0350131. doi: 10.1371/journal.pone.0350131 (PMC13218492; doi:10.1371/journal.pone.0350131)
Supplement: Attachment — Submitted filename: Review_PONE-D-25-68551.pdf [file pone.0350131.s002.pdf]

Dear Authors,

I have read your manuscript with great interest. I want to congratulate the authors on a well-conducted study. The study can constitute an important contribution to the empirical evidence on peer violence following the Socio-Ecological Model and the need for school and community-level initiatives to mitigate adolescent violence. However, I would like to draw the authors' attention to aspects of their manuscript that would need more detailed elaboration and consideration.

Methods section

- Lines 190 – 198: Please add references to each measure to support your claim that *"To facilitate interpretation relative to reference groups in the logistic regression analysis, all quantitative independent variables were categorized."*
- Lines 131-134: Please change your wording from ACEs to Household Challenges ACEs, as you do include any other variables that examine other ACEs (e.g., child maltreatment), rather than divorce/separation. It might give the impression that you will examine a whole range of ACEs that are not actually included in the analyses. Please also see my comment on the discussion section.

Discussion section

- Line 287: Please be accurate when using terms like 'rates' to refer to proportions that do not include time in the calculations.
- Lines 296-298: Please elaborate further on this sentence. As it is written, one might think that adolescent victims of violence have complete agency to deter violence if they have high self-efficacy levels. This statement does not account for other factors that are certainly present among victims of peer violence. Please add references accordingly and explain your claim.
- Throughout your discussion section, please add a subsection for each level of the Socio-Ecological Model that mentions other types of adversity, in particular childhood adversity (ACEs). For example, when explaining the results of non-intact parental status, you could add a brief overview of how parental divorce/separation exerts a toll on effective parenting strategies and how this is associated with adolescent development. This will strengthen your claim that, by examining only one household challenge, ACEs of divorce, your study provides sufficient evidence to act on one-parent households. Also, you might want to mention how other household challenges, such as household contact with the police and substance use, alongside divorce, could be associated with peer violence.
- Lines 353-354: Please explain the phrase '*"one-size-fits-all" approach to school discipline is insufficient,'* and provide references to previously examined interventions or services.
- Lines 345-349: Please do not overstate your conclusions. Your study examined one factor in each level of the Socio-Ecological Model. It might be best to be specific and then, in future directions, add the variables that were not examined in this study, acknowledging that peer violence is a complex public health issue that requires examination of several factors at each level of the Socio-Ecological model.

- Line 375: Please add how other studies examining adversity, either in childhood or in adulthood, could further help explain peer violence. Overall, provide more information about the future direction section, including the methodology, variables to be included, and conceptual frameworks.
