# Supplementary material for: Individual, community, and societal drivers of adolescent peer violence in Asella, Ethiopia: A school-based cross-sectional study utilizing the socio-ecological model
Source: PLoS One. 2026 May 28;21(5):e0350131. doi: 10.1371/journal.pone.0350131 (PMC13218492; doi:10.1371/journal.pone.0350131)
Supplement: Attachment — Submitted filename: REVIEWERS COMMENT.pdf [file pone.0350131.s003.pdf]

REVIEWERS COMMENT

Individual, Community, and Societal Drivers of Adolescent Peer Violence in Asella, Ethiopia: A School-Based Cross-Sectional Study Utilizing the Socio-Ecological Model

Please effect the corrections as shown in the tracked comments. Specifically, the following should be carried out:

1. In the abstract section, please include arguments that demonstrate the urgency and novelty of the study. The abstract is too dense with technical terms such as "Socio-Ecological Model (SEM)," "General Self-Efficacy (GSE)," and "bully-victims" without brief explanations, which can confuse the general reader.
2. The introduction presents issues that are too general and lack specificity regarding peer violence in urban settings like Asella. For example, there is no clear distinction between peer violence and other forms of violence, such as gender-based violence (GBV). Furthermore, there is no clear emphasis on urgency: no real-time data are available in Ethiopia, which would strengthen its relevance. The introduction may also be enhanced with clearer contextualization within secondary school education. The explanation of the underlying theory (Socio-Ecological Model) could be more systematic, and the articulation of research gaps could be made more explicit.
3. In the methods section, the validity and reliability of the research instrument are not described in sufficient detail. In addition, the data collection procedures require further clarification. Inclusion criteria remain unclear; specify the exact age range and grade for participants to avoid selection bias, as older adolescents may have increased exposure to violence. Prevalence assumptions for sample size calculations are drawn from non-specific peer violence studies.
4. In the results section, please add narrative explanations for each table. The presentation of the results remains largely descriptive and lacks sufficient interpretive depth. The results do not display crude OR for comparison with adjusted OR (AOR), making it difficult to assess confounding factors.
5. The discussion section is too concise and normative, more like an extension of the conclusion than an in-depth analysis. The lack of exploration of causal mechanisms (e.g., how low self-efficacy mediates the effects of witnessing violence) makes it less compelling. Besides that, the linkage to the socio-ecological model needs to be strengthened. In addition, comparisons with previous studies are limited, and the implications for school health education are not discussed in sufficient depth.
6. In the conclusion section, please add research limitations.
