# Supplementary material for: Individual, community, and societal drivers of adolescent peer violence in Asella, Ethiopia: A school-based cross-sectional study utilizing the socio-ecological model
Source: PLoS One. 2026 May 28;21(5):e0350131. doi: 10.1371/journal.pone.0350131 (PMC13218492; doi:10.1371/journal.pone.0350131)
Supplement: Attachment — Submitted filename: Response_to_Reviewers_PONE-D-25-68551.pdf [file pone.0350131.s004.pdf]

Response to Reviewers

Manuscript ID: PONE-D-25-68551

Title: Individual, Community, and Societal Drivers of Adolescent Peer Violence in Asella, Ethiopia: A School-Based Cross-Sectional Study Utilizing the Socio-Ecological Model.

Dear Dr. Bloom and Reviewers,

We would like to thank the editorial team and the reviewers for their thoughtful and constructive feedback on our manuscript. We have carefully addressed each point raised to improve the clarity, technical rigor, and interpretive depth of our study. Below is our point-by-point response.

Part 1: Responses to Journal Requirements

1. Funding Statement and Financial Disclosure

- **Journal Request:** Clarify sources of funding and the role of the funder.
- **Author Response:** We have updated our Funding Statement to be more explicit. We confirm that this research was financed by Arsi University, which provided financial support for data collection and field per diems. The funder had no role in study design, data collection and analysis, decision to publish, or preparation of the manuscript. As requested, all funding-related text has been removed from the "Acknowledgments" section of the manuscript.

2. Data Availability and Anonymization

- **Journal Request:** Remove identifying data from the supporting information file.
- **Author Response:** We have fully anonymized the dataset. All potentially identifying information, including town names, specific dates, and non-random ID numbers, has been removed or replaced with random identifiers to protect participant privacy in accordance with PLOS ONE policy. The revised file <Anonymized_Data_Set.csv> has been re-uploaded.

3. Figure and Table Citations

- **Journal Request:** Ensure Figure 1 and Tables 2, 3, and 4 are referred to in the text.
- **Author Response:** We have ensured that Figure 1 and all tables (2, 3, and 4) are now explicitly cited in the relevant sections of the manuscript to guide the reader.

Part 2: Responses to Reviewer #1

1. Categorization of Variables (Lines 163-173)

- **Reviewer Comment:** Please add references to support the categorization of quantitative independent variables.
- **Author Response:** We have added peer-reviewed references to the Methods section to support our approach to categorizing these variables **(Lines 163-173)** for logistic regression analysis, ensuring consistency with established public health research methodologies.

2. Terminology Change: ACEs (Lines 150-151)

- **Reviewer Comment:** Change wording from "ACEs" to "Household Challenges" to be more specific to the variables analyzed (e.g., divorce/separation).
- **Author Response:** We have adopted the term "Household Challenges (HHCs)" throughout the manuscript to accurately reflect that our study specifically examined divorce and separation rather than the full spectrum of childhood maltreatment.

Part 3: Responses to Reviewer #2

1. Abstract Clarity and Novelty

- **Reviewer Comment:** Include arguments for urgency/novelty and define technical terms like SEM and GSE.
- **Author Response:** The abstract has been revised to emphasize the novelty of providing real-time data on school-based peer violence in Ethiopia. We have also added brief definitions for the Socio-Ecological Model (SEM) and General Self-Efficacy (GSE) to improve accessibility for a general audience.

2. Introduction Specificity

- **Reviewer Comment:** Provide a clearer distinction between peer violence and GBV and emphasize the lack of real-time data in Ethiopia.
- **Author Response:** We have added a dedicated paragraph in the Introduction (Lines 89-98) that clarifies the conceptual differences between peer violence and GBV. We have also emphasized the scarcity of contemporary, school-based data in urban Ethiopia, highlighting the novelty of our study in addressing this specific research gap.

3. Methods: Validity, Reliability, and Inclusion Criteria

- **Reviewer Comment:** Describe instrument validity/reliability in detail and specify the exact age range and grade for participants.
- **Author Response:** We have significantly expanded the description of our methodology to address this comment.

- **Inclusion Criteria:** We have explicitly stated that the study included adolescents in **Grades 9–12 (aged 15–19)** to ensure a developmentally appropriate and consistent sample.

Validity and Reliability: Table 1, which details the measurement instruments and their psychometric properties. We now report high internal consistency for the violence scales (Cronbach’s α=0.83) and the GSE scale (α>0.70), along with test-retest reliability scores. We

have also clarified the translation and pre-testing procedures used to ensure face and content validity in the Ethiopian context.

4. Results: Interpretation and Odds Ratios

- **Reviewer Comment:** Add narrative explanations for tables and display Crude OR (COR) alongside Adjusted OR (AOR).
- **Author Response:** We have updated Table 4 to present both **Crude Odds Ratios (COR)** and **Adjusted Odds Ratios (AOR)** as requested. This allows for a transparent comparison of how variables behave before and after controlling for confounders. Additionally, we have included a comprehensive narrative explanation in the Results section (Lines 308-325) that interprets these findings through the different levels of the Socio-Ecological Model.

5. Discussion: Causal Mechanisms and SEM Linkage

- **Reviewer Comment:** Explore causal mechanisms and strengthen the linkage to the SEM.

- **Author Response:** We have added a synthesis paragraph in the Discussion (Lines 378-391 titled "Synthesis of Causal Mechanisms within the SEM." This section explores the "cascading risk" between societal poverty, community exposure, and individual self-efficacy, providing a clearer theoretical explanation of how these levels interact to drive adolescent violence in Asella.

- **6. Conclusion: Limitations**

- **Reviewer Comment:** Please add research limitations.
- **Author Response:** We have expanded the "Strengths and Limitations" section to provide a more critical evaluation of the study's scope. We have explicitly addressed the **cross-sectional nature** of the data, the potential for **social desirability bias**, and the **geographic focus** on urban schools in Asella, which may limit generalizability to rural contexts. We also acknowledged the exclusion of **cyber-bullying** to clarify the study's focus on school-based physical and relational aggression.
